# Supplementary figures and images for: Hammerhead-type FXR agonists induce an enhancer RNA Fincor that ameliorates nonalcoholic steatohepatitis in mice
Source: eLife. 2024 Apr 15;13:RP91438. doi: 10.7554/eLife.91438 (PMC11018349; doi:10.7554/eLife.91438)

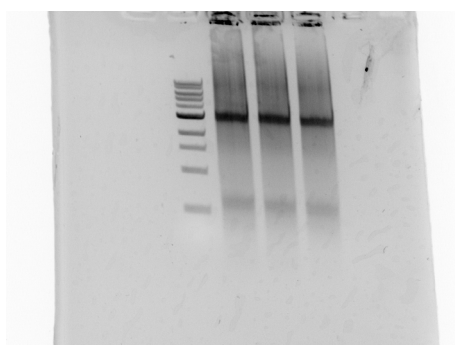

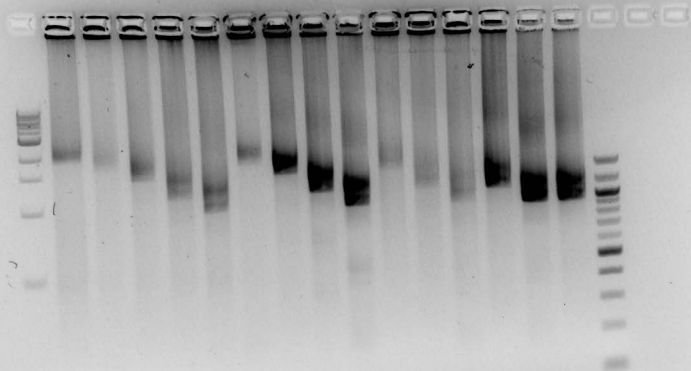

Supplement: Figure 3—source data 1. [file elife-91438-fig3-data1.zip › Figure 3-source data 1/Figure 3-source data 1.pdf]

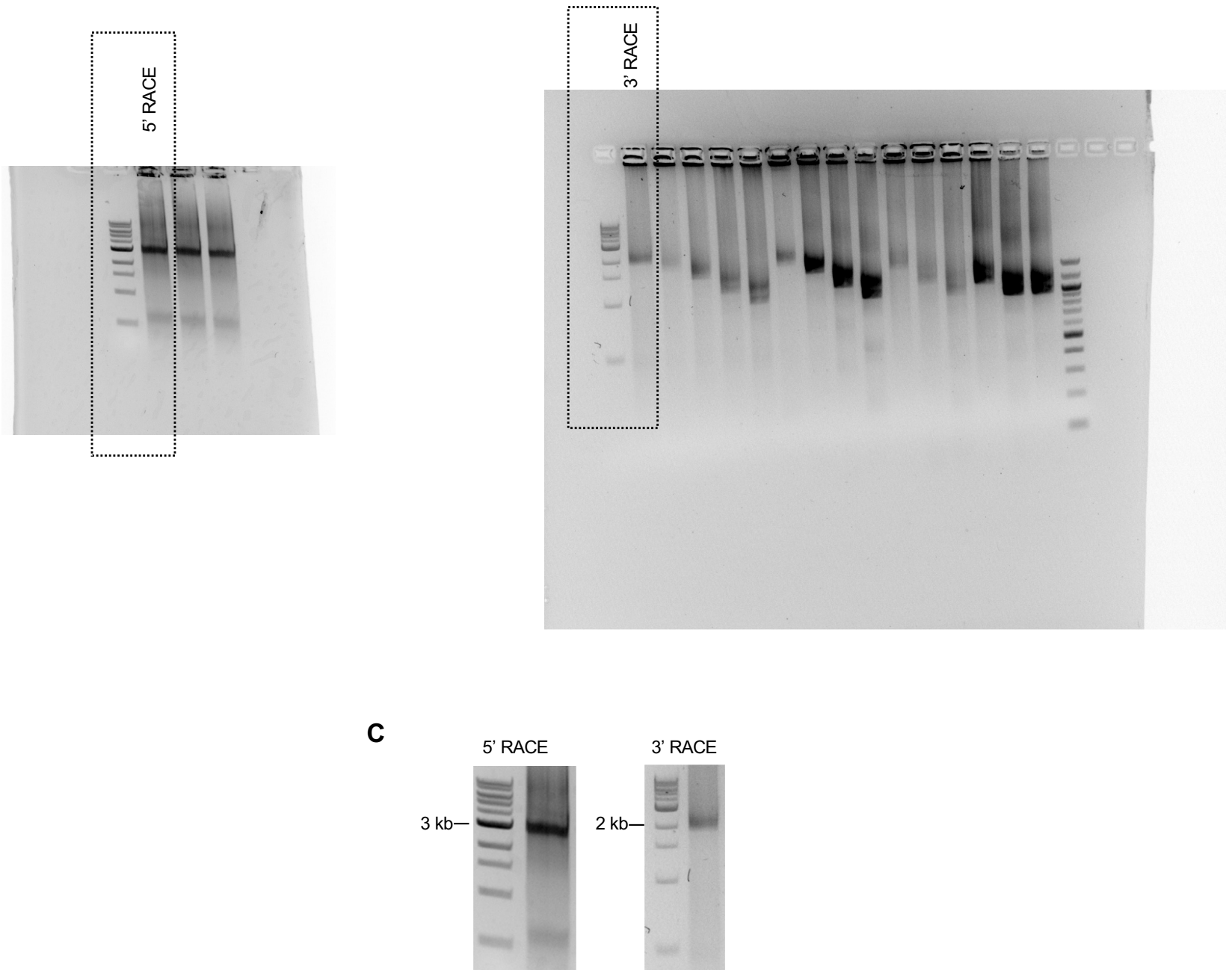

Figure 3

Supplement: Figure 3—source data 2. [file elife-91438-fig3-data2.zip › Figure 3-source data 2/Figure 3-source data 2.pdf]

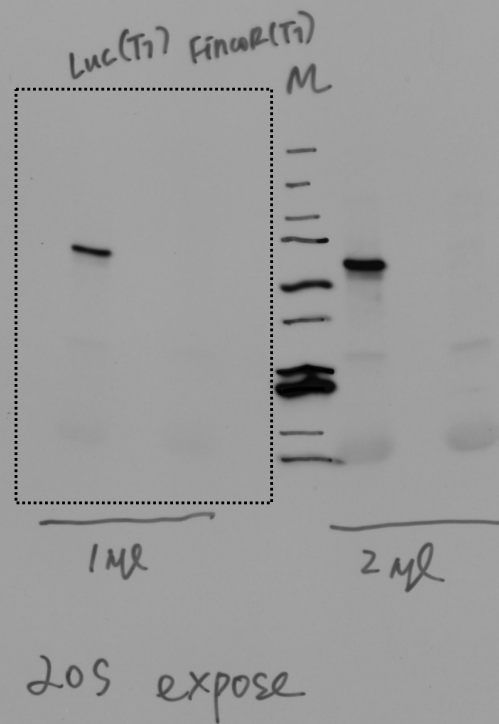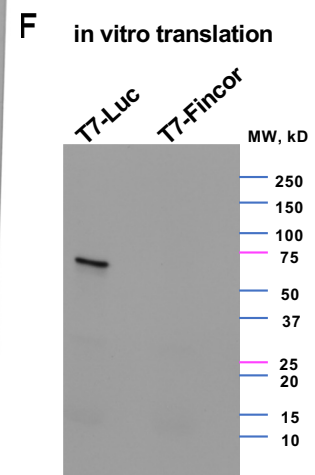

Figure 3

Supplement: Figure 3—source data 4. [file elife-91438-fig3-data4.zip › Figure 3-source data 4/Figure 3-source data 4.pdf]

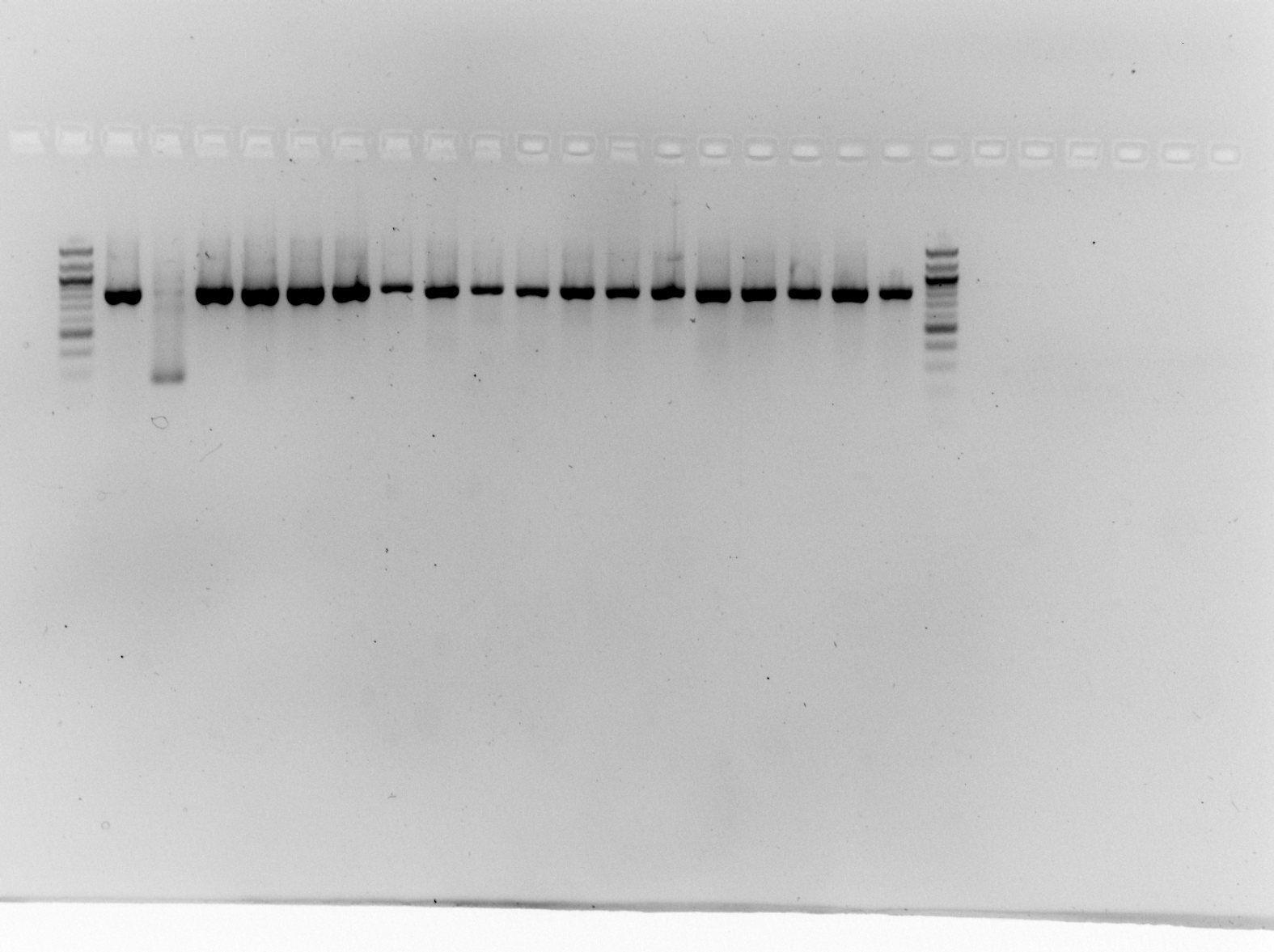

Supplement: Figure 5—figure supplement 1—source data 1. [file elife-91438-fig5-figsupp1-data1.zip › Figure 5-figure supplement 1-source data 1/Figure 5-figure supplement1-source data 1.tif]

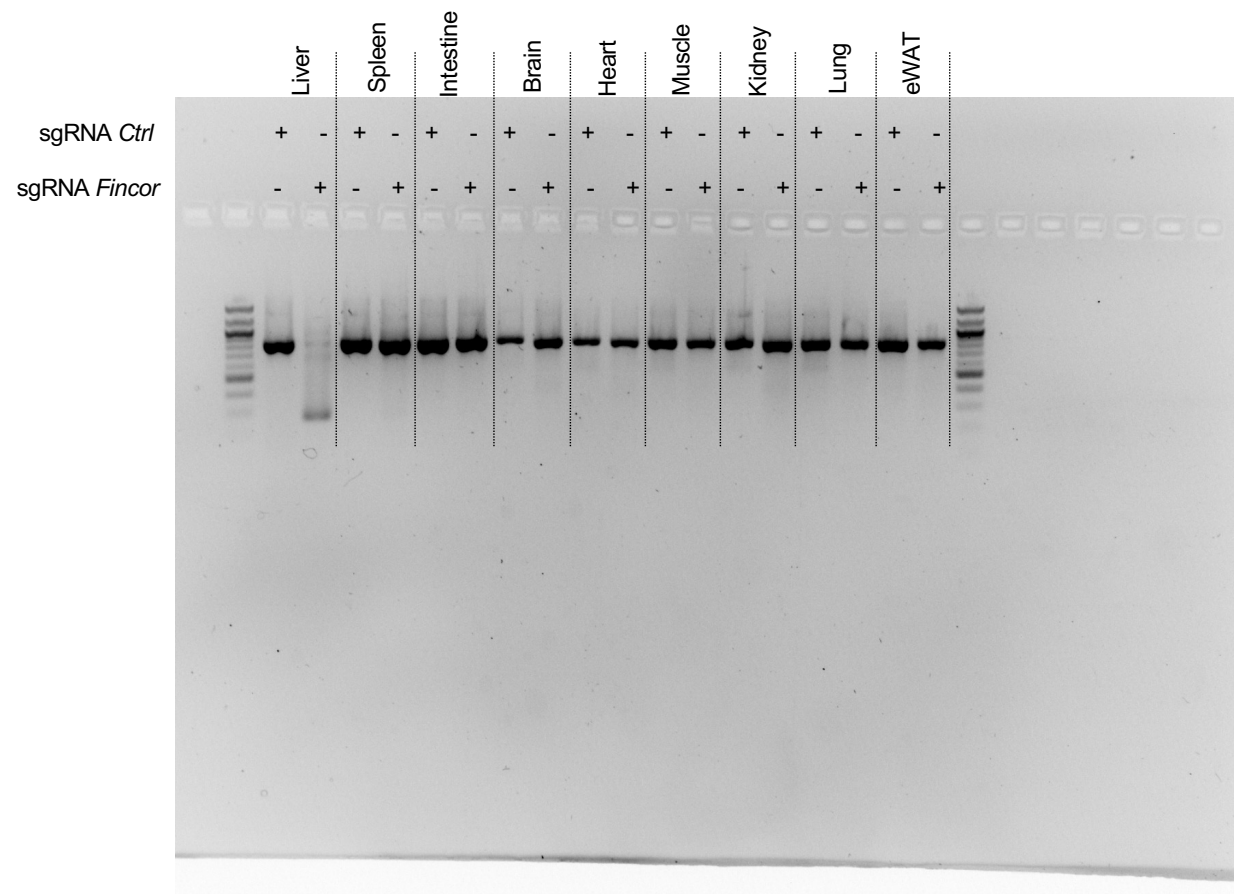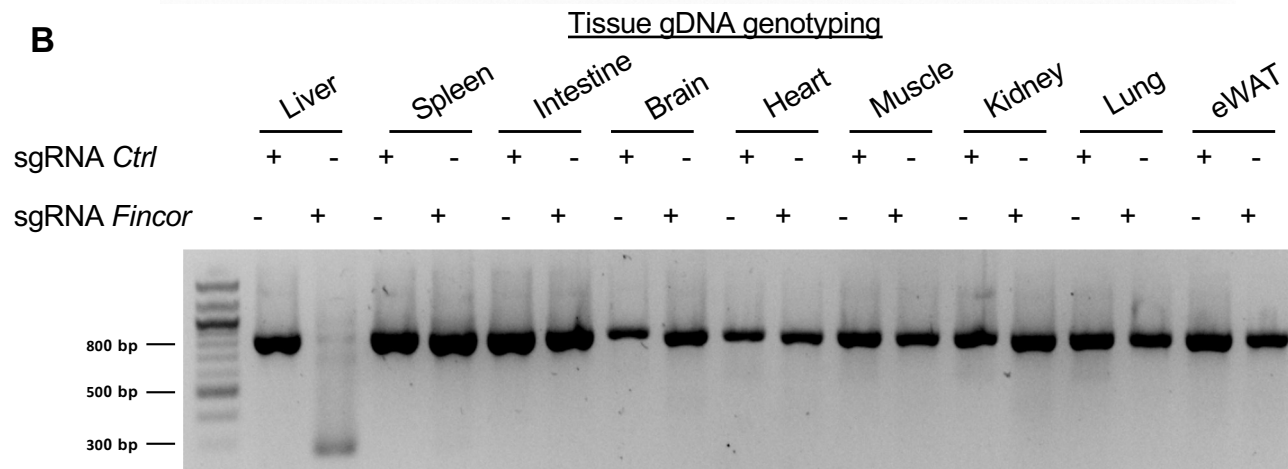

Figure 5-figure supplement 1

Supplement: Figure 5—figure supplement 1—source data 2. [file elife-91438-fig5-figsupp1-data2.zip › Figure 5-figure supplement 1-source data 2/Figure 5-figure supplement1-source data 2.pdf]
