# Supplementary material for: Hammerhead-type FXR agonists induce an enhancer RNA Fincor that ameliorates nonalcoholic steatohepatitis in mice
Source: eLife. 2024 Apr 15;13:RP91438. doi: 10.7554/eLife.91438 (PMC11018349; doi:10.7554/eLife.91438)
Supplement: Supplementary file 3. [file elife-91438-supp3.docx]

**Supplementary File 3a. Hepatic genome browser tracks of FXR, RXRα, LXR, PPARα, and HNF4α binding peaks at the *Fincor* locus.**

**Supplementary File 3b. PPARα occupancy in the *FincoR* enhancer region.** C57BL/6 male mice were fasted overnight with or without refeeding for 3 h and then sacrificed. ChIP assays were performed in liver samples to detect PPAR𝛂 occupancy at the FXR binding peak region close to the transcription start site of *FincoR*.
