# Supplementary material for: Hammerhead-type FXR agonists induce an enhancer RNA Fincor that ameliorates nonalcoholic steatohepatitis in mice
Source: eLife. 2024 Apr 15;13:RP91438. doi: 10.7554/eLife.91438 (PMC11018349; doi:10.7554/eLife.91438)
Supplement: Supplementary file 4. [file elife-91438-supp4.docx]

**Supplementary File 4: Predicted RNA binding proteins (RBPs) binding to *Fincor***

| RBP | Length | Motif | Domain | number of binding sites |
| --- | --- | --- | --- | --- |
| KHDRBS1 | 7 | UAAAAAG | KH | 1 |
| RBM38 | 7 | UUGUGUG, GUGUGUG | RRM | 3 |
| YBX2 | 7 | CACACCA | CSD | 11 |
| YBX3 | 7 | CACACCA | CSD | 11 |
| PCBP1 | 6 | CUUUCC | KH | 1 |
| KHSRP | 6 | UGCAUG | KH | 2 |
| PTBP1 | 6 | CUCUCU | RRM | 1 |
| PTBP2 | 6 | CUCUCU | RRM | 1 |
| 4KZD | 5 | GAAAC | N/A | 1 |
| 4KZE | 5 | GAAAC | N/A | 1 |
| 4Q9Q | 5 | GAAAC | N/A | 1 |
| DAZL | 5 | GUUCU | RRM | 6 |
| MSI1 | 5 | GUAGU | RRM | 1 |
| TLR3 | 5 | AAAGG | LRR;TIR | 12 |
