# Supplementary material for: Hammerhead-type FXR agonists induce an enhancer RNA Fincor that ameliorates nonalcoholic steatohepatitis in mice
Source: eLife. 2024 Apr 15;13:RP91438. doi: 10.7554/eLife.91438 (PMC11018349; doi:10.7554/eLife.91438)
Supplement: Supplementary file 5. [file elife-91438-supp5.docx]

Supplemental File 5. Primer sequences used in this study

Supplemental File 5a. List of primer sequences for mouse qRT-PCR

| **Gene** | **Forward Primer (5’-3’)** | **Reverse Primer (5’-3’)** |
| --- | --- | --- |
| *Fincor* | GCAAAGCACCTTCTAGCACA | GTCAGGGAGCTAACGAATGC |
| *Gcnt1* | GCATCGCATCCTGCTTTGATA | GGTCTGCCTTAACCCGACTC |
| *Nr0b2* | TCTGCAGGTCGTCCGACTAT | CAGGCAGTGGCTGTGAGAT |
| *Cyp7a1* | GGGATTGCTGTGGTAGTGAGC | GGTATGGAATCAACCCGTTGTC |
| *Cyp8b1* | CCTCTGGACAAGGGTTTTGTG | GCACCGTGAAGACATCCCC |
| *Srebp1c* | GCAGCCACCATCTAGCCTG | CAGCAGTGAGTCTGCCTTGAT |
| *Lpin1* | CATGCTTCGGAAAGTCCTTCA | GGTTATTCTTTGGCGTCAACCT |
| *Scd1* | TTCTTGCGATACACTCTGGTGC | CGGGATTGAATGTTCTTGTCGT |
| *Ifng* | GCG TCA TTG AAT CAC ACC TG | GAC CTG TGG GTT GTT GAC CT |
| *Ccl3* | TTCTCTGTACCATGACACTCTGC | CGTGGAATCTTCCGGCTGTAG |
| *Ccl2* | TTAAAAACCTGGATCGGAACCAA | GCATTAGCTTCAGATTTACGGGT |
| *Ccr2* | AAGAGGGCATTGGATTCACCACA | GCCGTGGATGAACTGAGGTAACA |
| *Lcn2* | GCAGGTGGTACGTTGTGGG | CTCTTGTAGCTCATAGATGGTGC |
| *Col1a1* | ATCGGTCATGCTCTCTCCAAACCA | ACTGCAACATGGAGACAGGTCAGA |
| *Col1a2* | CCTTTGTCAGAATACTGAGCAGC | GTAACTTCGTGCCTAGCAACA |
| *Acta2* | TCGGATACTTCAGCGTCAGGA | GTCCCAGACATCAGGGAGTAA |
| *Eda2r* | CACACTGCATAGTCTGCCCTC | GCCTTCTGGACCCGATTGA |
| *Fndc1* | GGGAGACATGGCAAACCTGT | TGGTAGGAGAGTATGTGGTGG |
| *Ctsb* | TCCTTGATCCTTCTTTCTTGCC | ACAGTGCCACACAGCTTCTTC |
| *Ctss* | GAAGTACGGCGTCTCATCTGG | CATGCCCACTTGGTAGGTATG |
| *Bcl2* | GTCGCTACCGTCGTGACTTC | CAGACATGCACCTACCCAGC |
| *36b4* | CCCTGAAGTGCTCGACATCA | TGCGGACACCCTCCAGAA |
| XR_007061585.1 | TTGTCATCAAGCCCTGTTCA | TCTGCTTTGTCTGAGGACCA |

Supplemental File 5b. List of primers used for mutagenesis

|  | Forward Primer (5’-3’) | Reverse Primer (5’-3’) |
| --- | --- | --- |
| Mutation | GTGAATTCTCTCCTAGaTCcTTGACaTTCCAAGTCATGTAC | GTACATGACTTGGAAtGTCAAgGAtCTAGGAGAGAATTCAC |

Supplemental File 5c. List of primer sequences for mouse ChIP-qPCR

| **Gene** | **Forward Primer (5’-3’)** | **Reverse Primer (5’-3’)** |
| --- | --- | --- |
| *Fincor* | TCCTAGGTCTTTGACCTTCCAA | TGTGCCCACTGGTGAAATAG |
| Non-specific region | GTGTACACGCCCAAACTTGA | GTTCACCTCTCTAGCTCACCT |

Supplemental File 5d. List of primer used for RACE

|  | **Gene specific primer (5’-3’)** |
| --- | --- |
| 5’RACE | GATTACGCCAAGCTT GGGAACTCACGTCTGGAAGCAGAAGCTG |
| 3’RACE | GATTACGCCAAGCTT GGACACTCGGGAATTGACAGTGGGTCAGG |
| 3’RACE nest primer | GATTACGCCAAGCTT GTGGGGGATAGCACAAGCGCCAGGATGC |

Supplemental File 5e. List of primer used for genotyping

|  | **Forward Primer (5’-3’)** | **Reverse Primer (5’-3’)** |
| --- | --- | --- |
|  | GTGATTTCCTTTCCCACAGC | GTCAGGGAGCTAACGAATGC |
